# Factors Associated with Breastfeeding Initiation: A Comparison between France and French-Speaking Canada

**DOI:** 10.1371/journal.pone.0166946

**Published:** 2016-11-30

**Authors:** Lisa-Christine Girard, Sylvana M. Côté, Blandine de Lauzon-Guillain, Lise Dubois, Bruno Falissard, Anne Forhan, Orla Doyle, Jonathan Y. Bernard, Barbara Heude, Marie-Josephe Saurel-Cubizolles, Monique Kaminski, Michel Boivin, Richard E. Tremblay, EDEN Mother-Child Cohort Study Group

**Affiliations:** 1 School of Public Health, Physiotherapy, and Sports Science, University College Dublin, Dublin, Ireland; 2 Geary Institute for Public Policy, University College Dublin, Dublin, Ireland; 3 Department of Social and Preventive Medicine, Université de Montreal, Montreal, Canada; 4 Institute of Genetic, Neurobiological, and Social Foundations of Child Development, Tomsk State University, Tomsk, Russian Federation, Russia; 5 Research Unit on Children’s Psychosocial Maladjustment (GRIP), Université de Montreal, Montréal, Canada; 6 INSERM, UMR 1153, Epidemiology and Biostatistics Sorbonne Paris Cité Centre (CRESS), Early ORigin of the Child’s Health and Development Team (ORCHAD), Villejuif, France; 7 Paris Descartes University, Paris, France; 8 School of Epidemiology, Public Health and Preventive Medicine, Faculty of Medicine, University of Ottawa, Ottawa, Canada; 9 Université Paris-Saclay, Université Paris-Sud, UVSQ, CESP, INSERM, Paris, France; 10 School of Economics, University College Dublin, Dublin, Ireland; 11 INSERM UMR 1153- Obstetrical, Perinatal and Pediatric Epidemiology Research Team (EPOPé), Center for Epidemiology and Statistics Sorbonne Paris Cité, DHU Risks in pregnancy, Paris, France; 12 School of Psychology, Université Laval, Quebec, Canada; 13 Departments of Pediatrics and Psychology, Université de Montreal, Montréal, Canada; Nathan S Kline Institute, UNITED STATES

## Abstract

**Background:**

Breastfeeding is associated with multiple domains of health for both mothers and children. Nevertheless, breastfeeding initiation is low within certain developed countries. Furthermore, comparative studies of initiation rates using harmonised data across multiple regions is scarce.

**Objective:**

The aim of the present study was to investigate and compare individual-level determinants of breastfeeding initiation using two French-speaking cohorts.

**Methods:**

Participants included ~ 3,900 mothers enrolled in two cohort studies in Canada and France. Interviews, questionnaires, and medical records were utilised to collect information on maternal, family, and medical factors associated with breastfeeding initiation.

**Results:**

Rates of breastfeeding initiation were similar across cohorts, slightly above 70%. Women in both Canada and France who had higher levels of maternal education, were born outside of their respective countries and who did not smoke during pregnancy were more likely to initiate breastfeeding with the cohort infant. Notably, cohort effects of maternal education at the university level were found, whereby having ‘some university’ was not statistically significant for mothers in France. Further, younger mothers in Canada, who delivered by caesarean section and who had previous children, had reduced odds of breastfeeding initiation. These results were not found for mothers in France.

**Conclusions and Implications for Practice:**

While some similar determinants were observed, programming efforts to increase breastfeeding initiation should be tailored to the characteristics of specific geographical regions which may be heavily impacted by the social, cultural and political climate of the region, in addition to individual and family level factors.

## Background

Breastfeeding is a global issue impacting upon multiple aspects of health and development for both mother and infant [1]. Documented associations between breastfeeding and maternal and infant health include, but are not limited to, reduced risk of breast and ovarian cancer and postpartum depression for mothers, obesity and type II diabetes for both mothers and infants, and reduced respiratory illness, gastrointestinal diseases, enteritis and better cognitive development for infants [2]. Economic gains have been documented with an estimated 1,500 US dollars in annual savings per family [3]. Economic benefits to society through health care system savings as a result of the beneficial effects of breastfeeding are probable [4].

Recommendations for exclusive breastfeeding during the first six months and mixed breastfeeding up to two years have been put forth by agencies worldwide including the World Health Organization, the European Commission, and the Public Health Agency of Canada [5–7]. However, examination of breastfeeding initiation rates reveals variation across regions [8–10]. While certain determinants of breastfeeding initiation may be localized to cultural or geographical regions, the benefits of breastfeeding to all societies make gaining a better understanding of these factors, an important goal for research. As described by the WHO in 2003, both clinical and population-based research that focuses on findings pertaining to references for international growth are critical to long-term global goals of increasing breastfeeding engagement [11]. Research in this area has only begun to take an international approach towards meeting this goal [12–13].

### Breastfeeding Initiation in France and Canada

Despite global recommendations and studies supporting the benefits of breastfeeding [1,7], initiation rates in certain developed countries remains low. According to national census data, the rates of infants who were ever breastfed in France were among the lowest across European and North American countries, reported as less than 70% [8,14]. National breastfeeding initiation statistics collected in 1998, 2003, and 2010 were estimated at 52.5%, 62.6%, and 68.7% respectively [15–16], with notable differences between regions [17]. By 2011, data from the French Longitudinal Study of Children (ELFE), a population-based sample comprised of ~18, 000 families, revealed a promising rise to 70.5% of infants who were ever breastfed [18].

In comparison to France, 81% of infants were reported as having ever been breastfed in Canada in 2001 and this increased to almost 88% by 2010 [14]. However, province-to-province initiation rates in Canada vary widely, and higher rates of breastfeeding have been found in English-speaking provinces as compared to French-speaking provinces [19]. For example, breastfeeding initiation rates have been reported as 91.1% in Ontario and as low as 72% in Quebec [20–22]. These statistics suggest existing differences not only between countries but also between cultures within countries. Interestingly, the rates of breastfeeding initiation in Quebec were closer to the national rates in France suggesting possible cultural similarities influencing initiation rates between these two French-speaking cohorts.

In both developed and developing countries, numerous programmes to increase breastfeeding initiation have been implemented. These public health efforts are based on documented associations between breastfeeding and positive child development [10,12,23–27]. With the exception of one study that cluster-randomised a breastfeeding intervention programme [28], no randomised controlled trials have been conducted in this field due to ethical considerations, leaving open the possibility that self-selection into breastfeeding accounts for the observed associations. Specifically, the positive ‘effects’ of breastfeeding may be attributable to characteristics of the mother, such as higher education and healthier lifestyle choices, rather than the direct effect of breastfeeding per se. Thus it is important to understand the individual-level determinants implicated in breastfeeding initiation and whether there are consistencies across regions.

A review of studies examining individual-level factors associated with breastfeeding in high-income countries would suggest certain consistencies such as higher maternal education and maternal age at child birth [20,29–30]. However, the effects and direction of other determinants such as income, parity, mode of delivery, preterm birth, and ethnicity have been less clear across studies [13,31–34]. These differences may be attributable to interaction effects with determinants particular to specific cultures or regions.

### Objectives

Previous studies have mainly focused on examining the individual factors predictive of breastfeeding for women in one particular cohort, potentially grouped by race or ethnicity. Yet identifying specific factors to cohorts from different countries by direct comparisons may help in tailoring breastfeeding promotion programmes to the characteristics of mothers in a culturally relevant way. The aim of the present study was to examine possible differences in the determinants of breastfeeding initiation using two birth cohorts from developed countries in Canada (Québec) and France. We asked whether maternal, family and medical determinants previously identified within the literature would present differently across these two French-speaking cohorts.

## Methods

### Participants

Mothers were drawn from two separate cohorts; the Quebec Longitudinal Study of Child Development (QLSCD; Quebec) and the Étude des Déterminants pre- et postnatals précoces du développement et de la santé de l’ENfant (EDEN; France). The Quebec cohort originally included 2,223 mothers selected from the Quebec Birth Registry, who gave birth to singletons between 1997 and 1998. Exclusion criteria for the overall cohort included multiple births, residence in northern Quebec, Cree, Inuit regions, or Indian reserves, and mothers with missing gestational duration data. Sampling and stratification procedures are reported elsewhere [35]. All mothers had complete data on breastfeeding and were thus eligible for inclusion in this study. Missing data accounted for less than 1.8% on any individual factor examined in association with breastfeeding initiation resulting in 2,144 mothers who were included in the final analyses. Ethics approval was obtained from the Quebec Institute of Statistics’ Ethics Committee. All data were collected during home visits when the infant was five months through interviews and questionnaires. Informed written consent was given prior to assessment. Maternal characteristics are reported in Table 1.

**Table 1:** Maternal, Family and Medical Characteristics: EDEN (France) and QLSCD (Quebec)

	**EDEN (France) n = 1,797**	**QLSCD (Quebec) n = 2,144**	***p***
**Ever Breastfed (Yes):**	1312 (73.0%)	1544 (72.0%)	0.486
**Highest Maternal Qualification:**			≤ 0.001
No Diploma	129 (7.2%)	381 (17.8%)	
High School Diploma	705 (39.2%)	566 (26.4%)	
Postsecondary Diploma	392 (21.8%)	621 (29.0%)	
University Degree	571 (31.8%)	576 (26.8%)	
**Maternal Age:**			≤ 0.001
≤ 24 years	275 (15.3%)	470 (21.9%)	
25–29 years	628 (34.9%)	685 (31.9%)	
30–34 years	609 (33.9%)	706 (32.9%)	
≥ 35 years	285 (15.9%)	283 (13.3%)	
**Family Income:**			0.311
Level 1	287 (16.0%)	348 (16.2%)	
Level 2	537 (29.9%)	602 (28.1%)	
Level 3	478 (26.6%)	550 (25.7%)	
Level 4	495 (27.5%)	644 (30.0%)	
**Mother Returned to Work at 4/5 Months (Yes):**	631 (35.1%)	382 (17.8%)	≤ 0.001
**Maternal Smoking during Pregnancy (Yes):**	472 (26.3%)	540 (25.2%)	0.440
**Preterm Infant (Yes):**	97 (5.4%)	108 (5.0%)	0.612
**Delivery Mode (Caesarean):**	287 (16.0%)	340 (15.9%)	0.923
**First Child (Yes):**	576 (32.1%)	948 (44.2%)	≤ 0.001
**Maternal Country of Birth (Canada/France):**	1716 (95.5%)	1888 (88.1%)	≤ 0.001

Note: Levels of family income differ across cohorts. For Quebec, annual income is reported. Level 1 represents an income of less than $20,000 (Canadian dollars), level 2: $20,000–39,999, level 3: $40,000–59,999 and level 4: $60,000 and higher. For France, monthly income is reported. Level 1 represents an income of less than 1,500€ (Euro), level 2: 1,501–2,300€, level 3: 2,301–3,000€ and level 4: 3,001€ and higher. The percentages representing maternal country of birth is Canada in the Quebec cohort and France in the France cohort.

For the France cohort, 2,002 pregnant women who were 18 years or older and less than 24 weeks of gestation, were initially recruited from two university hospitals in France (Nancy and Poitiers) between 2003 and 2006. Exclusion criteria were previous diagnoses of diabetes, expecting multiple births, unable to read/write in French, or planning on moving outside the catchment area within the next three years. The complete study protocol is described elsewhere [36]. Only women who had complete data on the breastfeeding variable were eligible for inclusion in this study, resulting in a possible 1,891 women. However, due to missing data, which accounted for less than 2.6% on any individual factor examined in the regression model, only 1,797 women were included. Ethics approval was obtained from the Ethics Committee of Kremlin Bicêtre (CCPPRB) and written consent was collected from each mother during enrollment. All data were collected through medical records, face-to-face interviews and self completed questionnaires at 24 and 28 weeks gestation, immediately following delivery and when the cohort infant was four months. Maternal characteristics are reported in Table 1.

### Measures

#### Breastfeeding

Maternal breastfeeding initiation was dichotomized into a binary Yes/No variable. Breastfeeding was defined as having ‘ever breastfed’ the cohort infant, whereby mothers who simultaneously introduced or used complementary liquids or solid foods in addition to breastfeeding were categorized as having initiated breastfeeding. A retrospective question was used to determine breastfeeding status in the Quebec cohort when infants were five months old: “Did you breastfeed (including the milk that you express for one or more bottles)?” While these data were collected retrospectively, reliability of recall of breastfeeding engagement has been established [37]. In the France cohort, breastfeeding information was collected prospectively at discharge from hospital and when infants were four, eight, and 12 months asking mothers to report on all feeding practices. For reasons of comparability across cohorts, the present study used breastfeeding data collected at four months, which was dichotomized into having initiated breastfeeding with the cohort infant (yes/no).

### Breastfeeding Determinants

#### Quebec

Mothers reported on their highest level of education (equivalent to no diploma, high school diploma, postsecondary/some university, university degree), annual household income in the last 12 months (computed into quartiles, less than or equal to $20,000, $20,001–39,999, $40,000–59,999, $60,000 or more), maternal age (24 years or under, 25–29 years, 30–34 years, 35 years or over), and maternal country of birth (born in Canada yes/no). Mothers also reported on maternal smoking during pregnancy (yes/no), type of delivery (vaginal, caesarean), and preterm birth derived from gestational age (delivered prior to the start of the 37th week yes/no). Finally, mothers were asked if they were presently working (yes/no) and the rank order of the cohort infant (from which first child status was deduced).

#### France

Information pertaining to the mother’s highest level of education (equivalent to no diploma, high school diploma, post secondary/some university, university degree), monthly household income (computed into quartiles, less than 1,500€, 1,501–2,300€, 2,301–3,000€, 3,001€ and higher), maternal age (24 years and under, 25–29 years, 30–34 years, 35 years and over), maternal country of birth (born in France yes/no), maternal smoking during pregnancy (yes/no) and the number of previous births (from which first child status was deduced) were collected during pregnancy. From medical records, type of delivery (vaginal, caesarean) and preterm birth (yes/no) were extracted. Finally, mothers reported on current working status (yes/no) when the cohort infant was four months old.

### Statistical Analysis

Data from both cohorts were combined into one larger dataset for analysis purposes (*N* = 3,941). Bivariate analyses were conducted to examine potential differences in both initiation rates and determinants across groups of mothers (initiated breastfeeding, never breastfed), grouped by cohort (Quebec, France) using Pearson’s chi-square tests. Factors in the bivariate analyses were retained in the logistic regression model if they were statistically significant. The statistical threshold for alpha was set to *p* = 0.050. Next, a logistic regression model was estimated to examine the contribution of maternal, family and medical determinants for breastfeeding initiation and to examine possible differences between cohorts. Heterogeneity between cohorts was examined in the model through the inclusion of cohort/predictor interaction terms. As some interaction terms were significant (i.e., maternal education, maternal age, type of delivery, and first child status) the analyses were also stratified by cohort. Linear contrasts were also tested for categorical variables to allow for a more in-depth understanding of any potential linear effects. In light of having prospective breastfeeding initiation data for mothers in EDEN, a sensitivity analysis was run to examine possible differences between models using the retrospective variable at four months as compared to the prospective variable extracted from hospital records shortly after the birth of the cohort infant. Models were comparable suggesting recall at four months presented with limited recall bias in the EDEN cohort (Please see S1 Table). All statistical analyses were conducted using IMB SPSS Statistics Version 20.0 [38]. To note, we use the term significant to denote statistical significance from herein.

## Results

### Bivariate Level Analyses

For Quebec mothers, 72% reported having initiated breastfeeding with the cohort infant, falling below the national norm of 81.9% in 1998–1999 [39]. Rates of breastfeeding initiation for mothers in France were reported as 73%, above the nationally reported rate of 62.6% in 2003 [16]. Differences in breastfeeding initiation rates were not significant between cohorts (i.e., Chi-squared: *X*^2^ (1, *N* = 3,941) = 0.486, *p* = 0.486). For both cohorts we found significant differences between mothers who initiated breastfeeding with the cohort infant and those who had not with respect to maternal education, maternal age, family income, maternal smoking during pregnancy and maternal country of birth. In the Quebec cohort only, significant differences were also found for first child status, type of delivery and preterm delivery. Maternal return to work at four and five months was not significant in either cohort. Please see Table 2.

**Table 2:** Breastfeeding Initiation according to Maternal, Family and Medical Characteristics: EDEN (France) and QLSCD (Quebec)

	**EDEN (France)**			**QLSCD (Quebec)**		
**Predictor**	Never Breastfed	Breastfed	*p*	Never Breastfed	Breastfed	*P*
**Maternal Education:**			≤ 0.001			≤ 0.001
No Diploma	45.0% (58)	55.0% (71)		45.4% (173)	54.6% (208)	
High School Diploma	34.6% (244)	65.4% (461)		32.2% (182)	67.8% (384)	
Some University	22.4% (88)	77.6% (304)		28.0% (174)	72.0% (447)	
University Degree	16.6% (95)	83.4% (476)		12.3% (71)	87.7% (505)	
**Maternal Age:**			0.013			≤ 0.001
≤ 24 years	34.2% (94)	65.8% (181)		37.7% (177)	62.3% (293)	
25–29 years	24.4% (153)	75.6% (475)		28.3% (194)	71.7% (491)	
30–34 years	27.8% (169)	72.2% (440)		24.2% (171)	75.8% (535)	
≥ 35 years	24.2% (69)	75.8% (216)		20.5% (58)	79.5% (225)	
**Family Income:**			≤ 0.001			≤ 0.001
Level 1	37.3% (107)	62.7% (180)		36.8% (128)	63.2% (220)	
Level 2	32.2% (173)	67.8% (364)		29.9% (180)	70.1% (422)	
Level 3	23.8% (114)	76.2% (364)		29.3% (161)	70.7% (389)	
Level 4	18.4% (91)	81.6% (404)		20.3% (131)	79.7% (513)	
**Maternal Return to Work at 4/5 Months:**			0.080			0.148
Yes	27.9% (176)	72.1% (455)		31.2% (119)	68.8% (263)	
No	24.0% (226)	76.0% (717)		27.5% (475)	72.5% (1,254)	
**Maternal Smoking:**			≤ 0.001			≤ 0.001
Yes	35.6% (168)	64.4% (304)		41.3% (223)	58.7% (317)	
No	23.9% (317)	76.1% (1,008)		23.5% (377)	76.5% (1,227)	
**Type of Delivery:**			0.279			0.037
Vaginal	27.5% (415)	72.5% (1,095)		27.1% (489)	72.9% (1,315)	
Caesarean Section	24.4% (70)	75.6% (217)		32.6% (111)	67.4% (229)	
**Preterm Infant:**			0.326			0.032
Yes	22.7% (22)	77.3% (75)		37.0% (40)	63.0% (68)	
No	27.2% (463)	72.8% (1,237)		27.5% (560)	72.5% (1,476)	
**First Child:**			0.779			≤ 0.001
Yes	26.6% (153)	73.4% (423)		23.4% (222)	76.6% (726)	
No	27.2% (332)	72.8% (889)		31.6% (378)	68.4% (818)	
**Maternal Country of Birth:**			≤ 0.001			≤ 0.001
France or Canada	27.8% (477)	72.2% (1,239)		29.7% (561)	70.3% (1,327)	
Outside France or Canada	9.9% (8)	90.1% (73)		15.2% (39)	84.8% (217)	

### Multivariate Level Analyses

A test of the full logistic regression model against the constant only model was significant indicating an improved model fit in predicting breastfeeding status, *x*^2^ (29) = 333.81, *p* = < .001. The complete model is presented in Table 3. The first column presents the *interaction term* within the overall model when the two cohorts were combined into one model. Given the significant interaction effects in the model, we then present the results for France and Quebec separately in columns two and three.

**Table 3:** Factors Associated with Breastfeeding Initiation of the Cohort Infant using Logistic Modeling: EDEN (France) and QLSCD (Quebec)

**Predictor**	**Combined CohortInteraction Term N = 3,941**			**EDEN (France) N = 1,797**			**QLSCD (Quebec) N = 2,144**		
		Adjusted Odds Ratio	95% C.I		Adjusted Odds Ratio	95% C.I		Adjusted Odds Ratio	95% C.I
**Maternal Education:**									
University Degree		ref	ref		ref	ref		Ref	ref
Some University		1.85	1.17–2.94		0.75	0.54–1.05		0.41	0.29–0.56
High School Diploma		1.22	0.77–1.93		0.45	0.33–0.62		0.37	0.26–0.52
No Diploma		1.17	0.63–2.16		0.28	0.18–0.46		0.24	0.16–0.35
**Maternal Age:**									
≥ 35 years		ref	ref		ref	ref		Ref	ref
30–34 years		1.07	0.66–1.75		0.82	0.59–1.15		0.77	0.54–1.09
25–29 years		1.75	1.06–2.88		1.15	0.81–1.63		0.67	0.46–0.94
≤ 24 years		0.57	1.17–3.72		1.19	0.78–1.82		0.57	0.39–0.85
**Family Income:**									
Level 4		ref	ref		ref	ref		Ref	ref
Level 3		0.92	0.60–1.43		0.87	0.63–1.21		0.95	0.71–1.27
Level 2		0.64	0.40–1.01		0.70	0.50–0.97		1.09	0.80–1.48
Level 1		0.76	0.43–1.32		0.65	0.43–0.99		0.86	0.60–1.25
**Smoking Status:**									
Smoker		1.16	0.83–1.62		0.77	0.59–0.97		0.65	0.52–0.82
**Type of Delivery:**									
Caesarean		1.83	1.22–2.75		1.21	0.89–1.64		0.66	0.50–0.86
**Preterm Infant:**									
Yes		1.87	0.96–3.63		1.25	0.75–2.09		0.67	0.44–1.04
**First Child Status:**									
Yes		0.57	0.41–0.80		0.95	0.74–1.21		1.67	1.34–2.08
**Maternal Country of Birth:**									
Outside Canada or France		1.76	0.75–4.11		4.05	1.90–8.63		2.31	1.57–3.39
**Cohort:**									
EDEN		0.71	0.43–1.22						
Constant:		7.97			5.66			5.24	
Nagelkerke Pseudo *r*^2^:	.119			.090			.139		

Note: Family income assessments were not directly comparable so quartiles of income to allow for comparison across cohorts were created.

For maternal country of birth the reference categories were Canada for QLSCD and France for EDEN. The first column in the table represents the interaction term when both cohorts were combined into one model, not the coefficient of the combined cohorts, followed by the results for each cohort separately. The Nagelkerke Pseudo *r*^2^ is reported as a measure of model fit where the likelihood values range between 0 and 1.

Maternal education was significantly associated with breastfeeding initiation for both cohorts where mothers with a university degree were more likely to initiate breastfeeding as compared to mothers who had no diploma. The post hoc linear contrast of maternal education revealed a significant trend, Chi-squared test for linear trend: *X*^*2*^(1, 4,078) = 211.63, *p* = < .001, whereby each additional level of education was associated with an increased probability of breastfeeding initiation. Interestingly, the interaction effect between cohorts was significant. The difference between cohorts occurred at the level of ‘some university’ against having ‘a university degree’ only, whereby having some university was not significant for mothers in France. Mothers in both cohorts who abstained from smoking during pregnancy were more likely to breastfeed as compared to mothers who smoked during their pregnancy. Maternal country of birth was a significant determinant of breastfeeding initiation in both cohorts, whereby mothers born outside the cohort country were more likely to initiate breastfeeding as compared to native-born mothers. Finally, across both cohorts, family income and having a preterm infant were not found to be significant determinants of breastfeeding initiation.

Differences between cohorts were also observed. For example, maternal age was a significant determinant for Quebec mothers but not for mothers in France. The post hoc linear contrast for age, Chi-squared test for linear trend: *X*^*2*^(1, 2,222) = 33.00, *p* = < .001, revealed that as maternal age increased, so did the likelihood of breastfeeding initiation. Quebec mothers for whom this was their first child were more likely to initiate breastfeeding the cohort infant yet this association was not found for mothers in France. Finally, Quebec mothers who delivered by caesarean section were significantly less likely to initiate breastfeeding as compared to mothers who delivered vaginally.

## Discussion

### Determinants of Breastfeeding Initiation

Similarities of breastfeeding determinants were observed across both cohorts. At the individual level, maternal education, maternal country of birth and maternal smoking status during pregnancy were all significant determinants of breastfeeding initiation. That is, higher levels of education, being of immigrant status and not smoking during pregnancy increased the odds of breastfeeding initiation with the cohort infant. These findings are largely consistent with the literature suggesting the stability of these determinants over the past few decades [17,29,40]. Of interest, the effect of maternal education at the university level differed between the cohorts. Whereas mothers in the Quebec cohort with ‘some university’ were approximately half as likely to initiate breastfeeding as compared to mothers with a ‘university degree’, this difference was not significant for mothers in France. University level education with a resulting degree, but not without, predicted higher odds of breastfeeding initiation for these mothers. With respect to family and medical factors, neither income nor the cohort infant being born preterm were significant determinants of breastfeeding initiation for either cohort.

Our results also highlight differences between cohorts. At the maternal level, a positive linear trend of age was found for Quebec mothers. While age was not statistically significant for mothers in France, the comparison between cohorts revealed differing patterns of effect. Younger mothers in France (i.e., < 29 years) were more likely to breastfeed compared to Quebec mothers of the same age. The differing pattern of age in France may reflect an interaction between previous cultural influences and the more recently increased support for breastfeeding initiatives in Europe over the past decade. For instance, more frequent exposure to policy initiatives promoting breastfeeding at earlier ages may be positively impacting on young mothers’ views of breastfeeding behaviour within a traditionally conservative culture. This difference between cohorts given the time between data collection highlights the importance of breastfeeding initiation evaluation within a combined anthropologic/epidemiologic framework. A more in-depth examination of the age trend in relation to government promotion and policy changes surrounding breastfeeding support across different time periods and different geographical regions is thus warranted.

At the family level, significant differences were found for first child status such that having no previous children increased the odds of breastfeeding initiation for Quebec mothers but not for mothers in France. Parity status has produced very mixed findings in the literature [41] and the direction of influence presumably depends on other factors (e.g., previous support, previous success, additional young children in the home). More studies are needed however before firm conclusions can be drawn surrounding the mechanism of effect and why differences across cohorts are commonly observed. Finally, delivering by caesarean section significantly decreased the odds of initiating breastfeeding with the cohort infant for Quebec mothers only. It is unclear why type of delivery was a significant determinant in one cohort but not the other. It may be possible that complications during the delivery, differing practices between hospitals, or BFHI’s contributed to this difference.

While the cohorts were similar in design thus allowing for meaningful comparisons between them, it is possible that these observed differences in the determinants of breastfeeding are also a reflection of the differing maternal baseline characteristics between the cohorts. Significant baseline differences in maternal education, age, country of origin and first child status were found. For example, in Quebec there were a larger amount of young mothers, with no diploma, for whom this was their first child and for whom this was not their country of origin. These differences in sample characteristics may reflect the differing recruitment and eligibility criteria between cohorts (i.e., in France, mothers needed to be 18 years of age or older, able to read and write in French, and stable rather than transient in their geographical location to be eligible for inclusion). Thus, that maternal age and first child status in particular were significant determinants of breastfeeding initiation for the Quebec cohort only; these findings may be partially explained by differences in maternal baseline characteristics.

### The Social and Political Climate

Despite differences in the individual determinants of breastfeeding, the similarity of breastfeeding initiation rates across cohorts may reflect similar socio-cultural climates at the respective times of data collection. For example, breastfeeding promotion has received much recent global attention from agencies such as the WHO/UNICEF, leading to calls for strategic action plans such as government-level initiatives. However, response to these calls differs across regions. Prior to data collection in the Quebec cohort, breastfeeding promotion was placed on the National Public Health Priorities list as a result of the large decrease in initiation rates (down to just below 50%) in the early 1990’s [42]. In response, the Quebec National Health Priority 1997–2002 five year strategy guide [42] was put forth by the Minister of Public Health whereby central themes on the promotion of both breastfeeding initiation and duration were recognized. The goal of the five year strategy guide was for initiation rates to rise to 80% by 2002, with 60% and 30% of mothers continuing to breastfed at three and six months respectively. At this time, Quebec also implemented the very first Baby-Friendly Hospital Initiative (BFHI) in Canada in 1999, and by 2001 had emerged with the first provincial-level breastfeeding policy supporting breastfeeding initiation. Further, maternal follow-up post hospital discharge regarding mother’s medical status, nutritional counseling, breastfeeding support, psychological support, parental competence support and guidance on positive infant development, were part of the recommendations to be implemented over this five-year period [42]. Despite these critical advances, the longstanding culture of Quebec has been largely governed by conservative values (e.g., the acceptability of exposure of a women’s breast in public places). It has been argued that as a result, social stigma, particularly as it relates to breastfeeding outside the home, has impeded the social support felt by mothers resulting in lower province-wide breastfeeding rates [43], as compared to the Anglo-Saxon provinces in Canada.

In the decades prior to data collection in France, similarly low national breastfeeding rates were observed. By 2000, breastfeeding promotion was given national priority. However, despite this, France still lacked a national policy plan with recommendations that were inline with the WHO prior to 2007 [8]. While initiation rates are now growing, they were still arguably low in the early 2000’s, which may reflect the absence of a national strategy plan. It may also be the case that the culturally conservative historical value system in France, similar to that of Quebec, or the preceding socially motivated high reliance on wet nurses post world war II among the upper and middle classes [44] may have contributed to these lower initiation rates. Within these regional contexts, the results offer important baseline information, from both an epidemiological and anthropological framework, regarding the medical, individual, and family level characteristics that were instrumental determinants of breastfeeding initiation in Quebec and France just prior to two historically important national-level programme and policy actions.

### Limitations

While there are inherent strengths of this study (e.g., the use of two large French-speaking cohorts from different continents, comparable measures and data collection strategies), some limitations must be noted. First, data was collected in 1998 (Quebec) and in 2003 (France), thus may not be reflective of present day *initiation* rates. However, *determinants* may have remained similar, in particular at the individual and family level. Nevertheless, the findings in this study remain particularly important by providing a comparative tool for observing possible changes since the initiation of the first BFHI in Quebec (particularly at the medical level of determinants), since France put breastfeeding initiation on the list of national priorities, and since the WHO’s 2003 global mandate towards promoting breastfeeding worldwide. Second, while strategies for data collection allowed for meaningful comparisons given the similarities of maternal, family socio-demographic and medical factors collected, it should be noted that systematic cultural differences render exact comparison of certain predictor variables challenging. For instance, the education systems in France and Canada differ, thus finer categorization in particular, at the secondary school level was not possible.

### Conclusions

The current work sheds important insights for a better understanding of the determinants of breastfeeding initiation just prior to two periods in time when large public health efforts were being implemented for breastfeeding promotion in both Canada and France. Across two French-speaking cohorts, in different geographical locations, the strongest predictors were maternal education and country of birth, implying that both individual and cultural level determinants have exerted influential roles in breastfeeding initiation. These determinants appear stable given the results of studies with other cohorts [18,31,34]. In developed countries, programme initiatives to promote breastfeeding engagement may do well to uniformly target mothers with lower levels of education. Additionally, given the consistent negative association between smoking and breastfeeding initiation, additional support for mothers who smoke should be made a priority. Conversely, factors related to maternal age, type of delivery, and additional children were implicated in different ways for mothers in these two geographical regions. Regional or cultural differences between any developed countries will likely always persist. Thus, policy reform and public health efforts to increase breastfeeding behaviour in reaching the 2020 global target will need to be tailored in culturally appropriate ways rather than using a global ‘one size fits all’ approach. Additionally, follow-up to this study with an evaluation of change in determinants since breastfeeding was put on the national health priorities list in both Quebec and France is warranted. With respect to future studies that focus on gaining a better understanding of the direct longitudinal effects of breastfeeding on child development, a careful account of maternal characteristics and perinatal variables is urged given the strength of social determinants.

## Supporting Information

S1 Table Sensitivity Analysis: Breastfeeding Data Extracted from Hospital Records in the EDEN Cohort (DOCX)
