# Supplementary material for: Factors Associated with Breastfeeding Initiation: A Comparison between France and French-Speaking Canada
Source: PLoS One. 2016 Nov 30;11(11):e0166946. doi: 10.1371/journal.pone.0166946 (PMC5130209; doi:10.1371/journal.pone.0166946)
Supplement: S1 Table — (DOCX) [file pone.0166946.s001.docx]

| S1 Table. Sensitivity Analysis: Breastfeeding Data Extracted from Hospital Records in the EDEN Cohort | | | |
| --- | --- | --- | --- |
| EDEN (*N* = 1,797) | | | |
| Predictor |  | **aOR** | **95% C.I** |
| Maternal Education: |  |  |  |
| University Degree |  | ref | ref |
| Some University |  | 0.76 | 0.55 – 1.06 |
| High School |  | 0.46 | 0.34 - 0.64 |
| No Diploma |  | 0.29 | 0.18 – 0.46 |
| Maternal Age: |  |  |  |
| ≥ 35 years |  | ref | ref |
| 30-34 years |  | 0.81 | 0.58 – 1.13 |
| 25-29 years |  | 1.14 | 0.81 – 1.62 |
| ≥ 24 years |  | 1.20 | 0.78 – 1.83 |
| Family Income: |  |  |  |
| Level 4 |  | ref | ref |
| Level 3 |  | 0.89 | 0.64 – 1.23 |
| Level 2 |  | 0.71 | 0.51 – 1.00 |
| Level 1 |  | 0.65 | 0.43 – 0.98 |
| Smoking Status: |  |  |  |
| Smoker |  | 0.75 | 0.59 – 0.96 |
| Type of Delivery: |  |  |  |
| Caesarean |  | 1.18 | 0.87 – 1.60 |
| Preterm Infant: |  |  |  |
| Yes |  | 1.20 | 0.73 – 1.98 |
| First Child Status: |  |  |  |
| Yes |  | 0.97 | 0.76 – 1.24 |
| Maternal Country of Birth: |  |  |  |
| Outside of France |  | 3.62 | 1.76 - 7.45 |
| Constant |  | 5.46 |  |
| Nagelkerke Pseudo *r*^2^: | .870 |  |  |
